# Pressurized intraperitoneal aerosol chemotherapy (PIPAC) in patients with peritoneal surface malignancies (PSM): a prospective single-center registry study

**DOI:** 10.1007/s00432-022-04517-w

**Published:** 2022-12-13

**Authors:** B. Jansen-Winkeln, J. Eberth, Y. Moulla, M. Mehdorn, S. Niebisch, K. Schierle, H. Bläker, F. Lordick, I. Gockel, R. Thieme

**Affiliations:** 1 Department of Visceral, Transplant, Thoracic and Vascular Surgery, University Hospital Leipzig, Liebigstraße 20, 04103 Leipzig, Germany; 2 Institute of Pathology, University Hospital Leipzig, Leipzig, Germany; 3 Department of Oncology, Gastroenterology, Hepatology, Pulmonology and Infectious Diseases, University Cancer Center Leipzig (UCCL), University Hospital Leipzig, Leipzig, Germany; 4 University Cancer Center Leipzig, University Hospital Leipzig, Leipzig, Germany

**Keywords:** Peritoneal surface malignancies (PSM), Pressurized intraperitoneal aerosol chemotherapy (PIPAC), Palliative chemotherapy, Peritoneal cancer index (PCI), Quality of life, Patient safety

## Abstract

**Purpose:**

Pressurized intraperitoneal aerosol chemotherapy (PIPAC) is a new, palliative approach for patients with peritoneal surface malignancies (PSMs). Its main goals are to control symptoms and ascites. For this experimental procedure, treatment efficacy and patient safety need to be closely monitored.

**Methods:**

We performed a prospective registry study for patients with PSMs. Cisplatin (C) (7.5 mg/m^2^ body surface) and doxorubicin (D) (1.5 mg/m^2^) were administered laparoscopically via PIPAC.

**Results:**

Between November 2015 and June 2020, we recorded data from 108 patients and 230 scheduled procedures. Tumor burden, patient fitness, quality of life, operating time and in-hospital stay remained stable over consecutive procedures. We recorded 21 non-access situations and 14 intraoperative complications (11 intestinal injuries, and three aspirations while inducing anesthesia). Three or more previous abdominal surgeries or cytoreductive surgery (CRS) with intraperitoneal hyperthermic chemoperfusion (HIPEC) were risk factors for non-access and intestinal injuries (χ^2^, *p* ≤ 0.01). Five Grade IV and three Grade V postoperative complications according to the Clavien–Dindo Classification (CDC) occurred. Median overall survival was 264 days (interquartile range 108–586). Therapies were primarily discontinued because of death (34%), progressive (26%), or regressive (16%) disease.

**Conclusion:**

PIPAC is effective in stabilizing PSMs and retaining quality of life in selected patients. Earlier abdominal surgeries and CRS with HIPEC should be considered when determining the indication for PIPAC. Randomized controlled studies are needed to evaluate PIPAC’s therapeutic benefits compared to systemic chemotherapy (sCHT) alone.

**Trial registration:**

NCT03100708 (April 2017).

**Supplementary Information:**

The online version contains supplementary material available at 10.1007/s00432-022-04517-w.

## Introduction

Peritoneal cancer primarily occurs as mesothelioma or metastasis of mainly gastrointestinal or gynecologic primary tumors (Henderson et al. 2018; Lemmens et al. 2011; Raza et al. 2014; Thomassen et al. 2014). Its diagnosis is associated with a worse prognosis than metastases from other locations (Franko et al. 2016).

The standard therapy depends on the therapeutic intention. If it is curative, sCHT and/ CRS with HIPEC are considered. In a palliative situation, sCHT and a purely symptom-oriented “watch and wait” strategy are practicable options. The treatment decision depends on the peritoneal cancer index (PCI) according to Sugarbaker (Harmon and Sugarbaker 2005; Jacquet and Sugarbaker 1996), primary tumor spread, histological entity and classification, lymphatic and distant metastases, patient's symptom burden, age, and comorbidities.

PIPAC is a relatively new palliative treatment option for clearly defined patients with PSMs, usually in combination with sCHT (Solaß et al. 2014). The indication is a palliative therapeutic strategy aiming to alleviate symptoms and ascites. Before this, a curative therapeutic option on the one hand and distant metastases on the other hand must be ruled out.

Previously, our group has described the surgical technique and safety aspects regarding the patient as well as surgical staff (Gockel et al. 2018). During the PIPAC procedure, a chemotherapeutic agent is nebulized intraabdominally by a special pump. It is then applied in the abdominal cavity using two laparoscopic trocars. The procedure is usually repeated every six weeks.

Although sCHT is still the gold standard in treating PSMs, it is believed to have little effect because of the limited drug delivery into the peritoneal surface (Minchinton and Tannock 2006). Other established methods of (usually liquid and heated) intraperitoneal chemotherapy deliver lower local concentrations of the chemotherapeutic agent than PIPAC does (Davigo et al. 2020; Macrì et al. 2011; Nadiradze et al. 2019; Solaß et al. 2012; Tempfer 2015). With PIPAC, surgical trauma, complication rates, and length of stay are lower than those reported for CRS with HIPEC. Combined with sCHT, there are very few additional toxicities and systemic side effects (Odendahl et al. 2015; Robella et al. 2016). Therefore, the procedure is reported to be well tolerated even by patients with comorbidities and in the elderly (Kepenekian et al. 2022).

However, the role which PIPAC plays in PSM treatment is still poorly defined. Presently, there are no prospective long-term data on PIPAC’s effects on prognosis and histopathologic regression (Grass et al. 2017). The response rates of specific tumor entities to this therapy are also unclear. The most likely effect is an alleviation or stabilization of tumor-related symptoms, ascites, and quality of life (Odendahl et al. 2015).

Therefore, in November 2015, we initiated a monocentric registry study (NCT03100708) to prospectively assess patients’ safety in conjunction with perioperative morbidity and mortality, feasibility and effectiveness, prognostic effect, and impact on quality of life.

## Materials and methods

### Patients

Before undergoing their first PIPAC procedure, our local multidisciplinary tumor board discussed all patients, namely their PCI (as defined by a most recent and standardized diagnostic laparoscopy in our clinic), comorbidities, and preferences. A curative approach involving sCHT, CRS, and HIPEC was initially ruled out.

Inclusion criteria were histologically proven PSM and our multidisciplinary tumor board’s positive recommendation. Exclusion criteria were extraperitoneal distant metastases, and an European Organization for Research and Treatment (ECOG) performance status higher than 2. Deviating from the standard PIPAC inclusion criteria, we treated eleven patients with distant metastases. Three patients with pleural metastases underwent simultaneous pressurized intrathoracic aerosol chemotherapy (PITAC). The remaining distant metastases had been stable or regressive under sCHT, but the peritoneal metastasis (PM) was progressing and causing a severe ascites burden. We decided to attempt PIPAC in these particular cases.

Before each scheduled PIPAC procedure, we questioned patients about symptoms associated with PSMs, i.e., abdominal pain, nausea or emesis, dysphagia, and obstipation according to our structured interviews. We evaluated their Nutritional Risk Score (NRS), American Society of Anesthesiologists (ASA) classification, ECOG performance status, and quality of life using the Quality of Life Questionnaire Core 30 (QLQ-C30) by the European Organization for Research and Treatment of Cancer (EORTC). The latter is divided into 15 items including global health (QL2), physical functioning, fatigue, nausea/vomiting, pain, and appetite loss. The individual scores are linearly converted to a 0–100 scale (Aaronson et al. 1993).

According to our standardized PIPAC perioperative protocol, on the day of admission, as well as on the first and third postoperative days, we measured serum creatinine (µmol/L), and C-reactive protein (mg/L) (COBAS C-system 8000 and E-module; Roche Diagnostics, Mannheim, Germany), as well as leucocytes (10^9^/L) (Sysmex XN 9000 system; Sysmex Europe GmbH, Norderstedt, Germany) following the guidelines of the German Medical Association (Berlin, Germany). Serum creatinine values below 0.6 mg/L could not be quantified more precisely and were, therefore, set to 0.6 mg/L for statistical analysis.

All patients were thoroughly informed by an experienced surgeon and a medical oncologist about the PIPAC procedure and gave their written informed consent.

The study was conducted from December 2015 to June 2020 and registered under clinicaltrials.gov (NCT03100708), carried out in accordance with the Declaration of Helsinki, and approved by our local University of Leipzig ethics committee (No. of the approval: 106-16-14032016).

### Surgical procedure

As described by our group (Gockel et al. 2018, 2020) and others (Hübner et al. 2017; Nowacki et al. 2018), the PIPAC procedure was performed laparoscopically and under general anesthesia, strictly following our internal standard operating procedures (SOP). First, 12 mmHg capnoperitoneum was induced via a mini-laparotomy measuring 1–2 cm. A 12 mm trocar (Kii Fios Advanced Fixation; Applied Medical, Düsseldorf, Germany) with video optics was inserted into the abdominal cavity. Then, a second 5 mm trocar was installed under visual control. If ascites was visible, it was aspirated, and the ascites volume determined. Following our guidelines for diagnostic laparoscopy, the surgeon described accessibility to the abdomen as “access” or “non-access” and the PCI was recorded according to Sugarbaker. We also raised the modified Adhesion Score according to Coccolini et al. (2013).

Peritoneal biopsies (flank, upper and lower abdomen, right and left side) were taken, where accessible, in a standardized manner according to our protocol. They were sent for pathological analysis. All samples were fixed in 4% buffered formalin, embedded in paraffin, cut in 4 µm thick layers, and stained with hematoxylin and eosin (H&E) using an automated slide stainer (Sakura Tissue Tek Prisma, Tokyo, Japan). The relative area covered by tumor cells (in %) was determined by two experienced pathologists (KS and HB). The biopsy containing the most tumor cells was considered when assessing tumor changes between PIPAC procedures.

The micropump (Capnomed GmbH, Villingendorf, Germany) was inserted into the 12 mm trocar to perform the PIPAC procedure. Following our checklist, the injection pump (Medrad Arterion Mark 7, Leverkusen, Germany) was carefully connected, and all personnel left the operating room. The chemotherapy application was controlled with a footswitch and could be monitored through a window in the closed-off preparation room. The anesthesia was supervised by the responsible staff in the same way. They had the option to apply medication through two syringe pumps connected to the patient by long tubes. Chemotherapy was applied through the injection pump at a maximum pressure of 200 psi and a flow rate of 0.5 mL/min. Subsequently, the micropump vaporized the drugs inside the abdominal cavity. First, 7.5 mg/m^2^ body surface C in 150 mL 0.9% NaCl solution was insufflated followed by 1.5 mg/m^2^ D in 50 mL 0.9% NaCl. The constant pressure of the capnoperitoneum of 12 mmHg was scrutinized to monitor both, any aerosol escaping the abdomen, and the patient’s relaxation status. After 30 min, the aerosol was evacuated over a closed system in the clinic’s airwaste. The pump was removed, and the surgeons inspected the situs for bleeding or lesions. Finally, all trocars were removed and the fascia and skin were sewn. No abdominal drainage was inserted. All single-use products were disposed off, while multiple-use instruments were cleaned and sterilized.

After having been monitored for a few hours in the post-anesthesia care unit, patients were transferred to our ward, where any postoperative complications were documented prospectively according to the CDC (Dindo et al. 2004) and our protocol.

### Data management and statistical analyses

All data including demographic, clinico-pathological, oncological, intraoperative, and perioperative parameters were prospectively recorded and retrospectively analyzed.

We relied on our patient registry for follow-up, if the given patient stayed with us in treatment or died in our hospital. We also utilized our local cancer registry, whenever patients resided within the administrative district of Leipzig. For additional information about the disease course and survival data, we contacted each patient’s physician (e.g., general practitioner or oncologist).

Values are presented as number and percent, mean with standard error of the mean (SEM), or median with (interquartile) range. Whenever measurements were taken for each PIPAC procedure (e.g., body mass index), the mean or median for each patient was calculated first. The overall mean or median was determined by starting from this now independent sample. SEM and (interquartile) range were also calculated from the latter sample. Data evaluations and statistical analyzes were carried out using Excel (Version 2105; Microsoft Corp., Redmond, WA, USA) and SPSS (Version 27.0; IBM Corp., Armonk, NY, USA). Figures were created using Excel and PowerPoint (Version 2105; Microsoft Corp., Redmond, WA, USA).

## Results

### Patients’ characteristics

Our total patient cohort consisted of 108 subjects, 55 women and 53 men aged a median 60 years (interquartile range 53–69). Sixty-seven (62%) patients were diagnosed with synchronous PM or primary peritoneal cancer, while 41 (38%) patients were diagnosed metachronous PM (Table 1). Included were patients with PSMs of defined origins only. The largest subgroup consisted of 41 patients (38%) with gastric cancer, followed by 26 (24%) patients with colorectal cancer. Seventeen patients (16%) had a pancreatico-biliary (PB) primary tumor (*n* = thirteen pancreatic, three bile duct, and one gall bladder cancer). Nine women (8%) with a gynecological primary tumor (*n* = two breast, three ovary, and four uterine cancers) were included. The remaining 15 (14%) patients were combined as “others” [*n* = seven mesotheliomas, three pseudomyxoma peritonei, and five cancers of unknown primary (CUP)]. CUPs were histologically classified to be most likely upper gastrointestinal, colorectal, pancreatico-biliary, gynecological, or goblet cell associated (each once) (Supplementary Table 1).

**Table 1:** Characteristics of all included patients

Patient characteristics	Value
No. of patients	108
Sex	
Female	55 (50.9%)
Male	53 (49.1%)
Age [years]	60 (53–69)
ECOG performance status	1 (0–1)
ASA	3 (2–3)
NRS	2 (2–3)
QL2	50 (33–67)
Synchronous PM or PPC	67 (62%)
Metachronous PM	41 (38%)

Values are presented as number (%) or median (interquartile range)

Age in years

*ECOG* Eastern Cooperative Oncology Group, *ASA* American Society of Anesthesiologists, *NRS* nutritional risk score, *QL2* Global Health Status (quality of life questionnaire core 30 by the European Organization for Research and Treatment of Cancer), *PM* peritoneal metastasis, *PPC* primary peritoneal cancer

The median number of PIPAC procedures per patient was 2 (interquartile range 1–3) with an overall repeatability rate of 63%. Five patients had undergone one or more PIPAC procedures (*n* = one, one, two, three, and four, respectively) in a different hospital before being treated by another PIPAC at the University Hospital Leipzig.

Ninety-one (84%) patients had already undergone sCHT before their first PIPAC procedure, while an additional 13 (12%) patients began their sCHT simultaneously (between PIPAC cycles or no more than 30 days before their first PIPAC procedure). In total, 60 (54%) patients underwent PIPAC and sCHT concurrently. Forty-four (41%) patients had undergone sCHT in the past, but not during their PIPAC cycles, and four (4%) were not treated with sCHT. Reasons for this were the lack of an indication for pseudomyxoma peritonei (*n* = two) and the patient’s personal decision against the tumor board’s advice. In one patient, the scheduled beginning of sCHT after PIPAC was canceled because of the patient’s death.

Seventy-eight (72%) of all patients had their primary tumors resected before inclusion in this study. Twelve (11%) patients had also received CRS with HIPEC (Supplementary Table 2).

### Canceled procedures

230 scheduled PIPAC procedures in 108 patients were screened in this study resulting in 189 PIPAC procedures carried out in 82 patients.

**Fig. 1:**
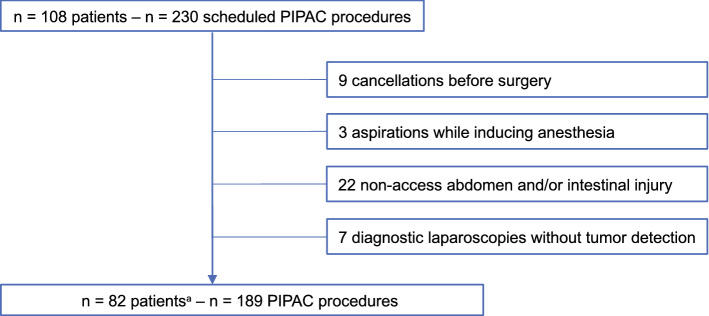
Flowchart of included patients and PIPAC procedures and reasons, why scheduled PIPAC procedures were not carried out. ^a^Patients with at least one successful PIPAC procedure. *PIPAC* pressurized intraperitoneal aerosol chemotherapy

Forty-one PIPAC procedures had to be canceled: In nine cases, patients were hospitalized for a planned PIPAC procedure but presented in a much worse condition during preoperative preparations, compared to their health status during preliminary examination (e.g., new distant metastases detected in a preoperative CT scan). Therefore, nine PIPAC procedures were rescinded before initiating surgery. In three instances, there were aspirations while inducing anesthesia. No chemotherapeutic agent was applied in seven diagnostic laparoscopies because no PSM was detected. Twenty-two PIPAC procedures could not be carried out because of a non-access abdomen and/or bowel lesions (Fig. 1).

### Preoperative symptoms and performance status

Upon preoperative physical examination and anamnesis (before each PIPAC), most patients presented none of the specifically assessed symptoms (abdominal pain, nausea or emesis, dysphagia, or obstipation). The remaining patients reported abdominal pain (55, 24%), nausea or emesis (47, 21%), obstipation (16, 7%), and/or dysphagia (4, 2%), with multiple answers possible. The more PIPAC procedures patients underwent, the fewer symptoms they reported (Supplementary Fig. 1).

Patients had a median ECOG performance status of 1 (interquartile range 0–1), an ASA class of 3 (interquartile range 2–3), and an NRS of 2 (interquartile range 2–3). The median global health score was 50 (interquartile range 33–67) (Table 1). None of the above-mentioned scores changed significantly (Analysis of Variance (ANOVA), *p* > 0.1) throughout multiple PIPAC procedures.

### Operative course

The mean duration of each PIPAC (104 ± 1.5 min.) remained unchanged with consecutive procedures. The median PCI evaluated during laparoscopy was 15 (interquartile range 6–24), while the median Adhesion score was 4 (interquartile range 0–12), and the median for maximum percentage of tumor cells in biopsies was 24% (interquartile range 5–60%) (Table 2). We identified no significant change (increase or decrease) in any of these parameters in conjunction with consecutive PIPAC procedures (ANOVA, *p* > 0.1), although on the one hand, a decreasing tendency became apparent in PCI and tumor cell count, and on the other hand an increase in adhesions. Measuring the ascites volume over multiple PIPAC treatments revealed a significant decrease for the first three PIPAC procedures (ANOVA, *p* = 0.016).

**Table 2:** Intraoperative findings

	1st PIPAC	2nd PIPAC	3rd PIPAC	4th PIPAC	5th PIPAC	6th PIPAC	7th PIPAC	8th PIPAC	9th PIPAC	10th PIPAC	11th PIPAC
No	77	48	27	14	8	6	3	3	1	1	1
Ascites (mL)	1946 ± 279	1319 ± 345	394 ± 205	607 ± 231	563 ± 334	533 ± 383	23 ± 12	300 ± 204	50	50	3500
PCI	17 (2–38)	15 (1–39)	14 (0–29)	18,5 (8–32)	14 (9–20)	16 (10–20)	13 (8–27)	17 (16–35)	22	17	28
Adhesion Score	3 (0–32)	3,5 (0–26)	7 (0–31)	6 (0–15)	0,5 (0–20)	2 (0–6)	2 (0–8)	2 (0–3)	2	n	n
Max. tumor cell proportion (%)	30 (0–95)	30 (0–90)	20 (0–95)	20 (0–70)	20 (0–95)	20 (0–25)	20 (1–30)	10 (1–30)	0	1	80
OP duration (min)	104 ± 2.4	103 ± 3.8	100 ± 3.4	91 ± 3.9	103 ± 4.7	92 ± 5.6	105 ± 8.5	114 ± 9.5	90	126	96
Post-OP in-hospital stay (days)	4 (2–18)	4 (1–28)	4 (2–5)	3,5 (2–6)	3 (2–5)	3,5 (2–5)	3 (3–3)	4 (3–5)	3	3	3

Values are presented as mean ± standard error of the mean (SEM) or median (range)

*PIPAC* pressurized intraperitoneal aerosol chemotherapy. Adhesion Score according to Coccolini, *PCI* peritoneal cancer index (according to Sugarbaker)

### Postoperative findings

The median postoperative in-hospital stay of four days (interquartile range 3–4) persisted across repeated procedures. The length of stay of sixteen patients was at or above the 95th percentile of seven days. Eight prolonged hospital stays were because of intraoperative complications.

Adverse events during PIPAC’s postoperative course (Grade II or above according to the CDC) occurred in twenty-seven out of 189 completed PIPAC procedures (14.3%), of which twenty-two (11.6%) were Grade II. Their incidence did not change significantly over repeated PIPAC procedures per patient. Four (2.1%) Grade IV adverse events occurred (*n* = three on 1st and one on 2nd PIPAC). One patient died after PIPAC. When taking all 213 procedures into account, during which patients were brought to the operating room for PIPAC, we documented 31 complications (10.8% Grade II, 2.4% Grade IV, and 1.4% Grade V). No Grade III events according to the CDC were recorded, since no postoperative interventions involving local or general anesthesia were performed outside an intensive care setting.

Mean values for leucocytes and serum creatinine always stayed within reference values. C-reactive protein concentrations also remained in a clinically unremarkable range (Table 3).

**Table 3:**
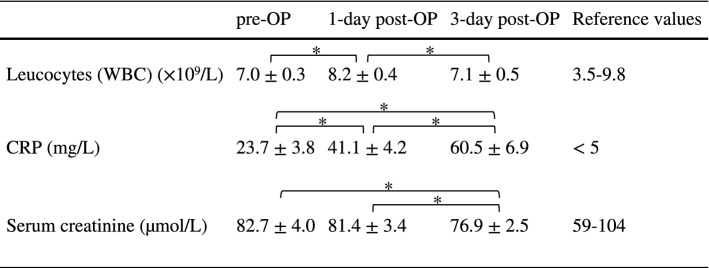
Hematology and clinical chemistry

Values are presented as mean ± standard error of the mean (SEM). Reference values according to the Institute for Laboratory Medicine, Clinical Chemistry and Molecular Diagnostics, University Hospital Leipzig, Leipzig, Germany

*WBC* white blood cells,*CRP* C-reactive protein,*OP* operative

*Significant change (Wilcoxon, *p* < 0.05)

### Safety

**Fig. 2:**
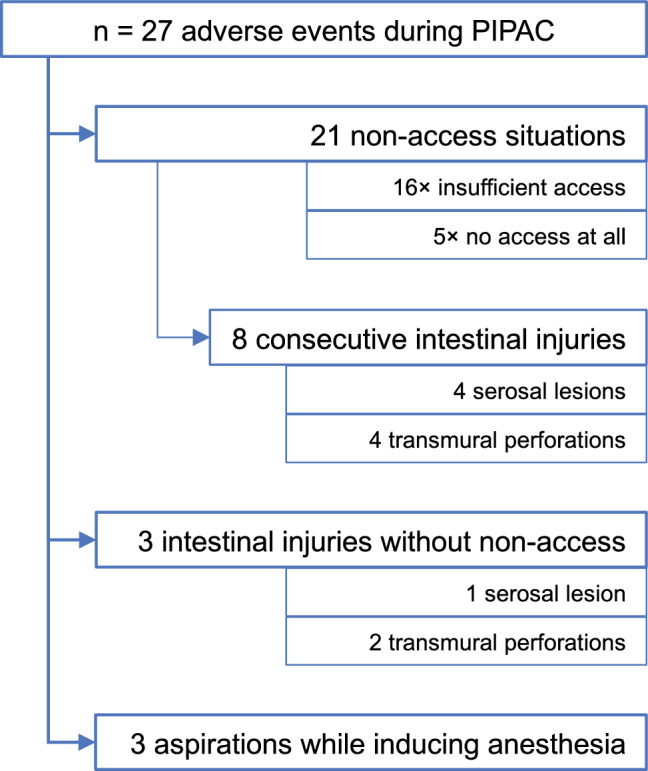
Number of adverse events during PIPAC, divided into subgroups. Number of patients and where their complications occurred. *PIPAC* pressurized intraperitoneal aerosol chemotherapy

During our study, we recorded 21 non-access situations (9.9% of all 213 procedures, during which patients were brought to the operating room for PIPAC) and 14 intraoperative complications (6.6%) (Fig. 2). The courses of treatments affected by intraoperative complications are described in detail.

#### Non-access

We identified 21 patients with a non-access abdomen, 15 during their first PIPAC attempt, and six during one of the subsequent ones. Five abdominal cavities were inaccessible, and 16 were inadequately accessible, as severe intraabdominal adhesions prevented establishing of a sufficient capnoperitoneum and would have obstructed the aerosol’s distribution. In eight of these twenty-one patients, inserting the trocar and adhesiolysis resulted in consecutive intestinal injuries, six of the small intestine, and two of the large intestine (each in equal parts serosa lesions and transmural perforations). The operation had to be converted to a laparotomy in five patients for an intestinal suture of these perforations.

The postoperative course of all non-access patients was mostly uneventful. Seventeen patients scored Grade 0 or I according to the CDC. The remaining four patients scored Grade II and are described briefly: One patient was given antibiotics (cefuroxime and metronidazole) after a non-access followed by an abscess in the abdominal wall. Three patients had to receive parenteral nutrition due to intestinal injury. One of them was also given somatotropin-inhibitory hormone (8 days) and octreotide (4 days) to control intestinal secretion and showed signs of a low-output fistula that had a stable flow rate of about 100 mL/d. Of the four patients, three were discharged in improved general condition. One patient developed a significant tumor progression during the hospital stay and was transferred to a hospice.

We attempted one second PIPAC procedure after a patient had presented with a non-access abdomen, which failed, because the abdomen remained inaccessible.

#### Intestinal injury without non-access

Three complications presented as lesions of the small intestine (*n* = one serosa lesion and two perforations) without major adhesions: Two of these were sewn and had complication-free peri- and postoperative courses. The remaining patient, however, experienced a more severe postoperative course in 2016. During his presurgical examination, he presented with stomachache, reflux, and a frequent gag reflex along with gastric outlet obstruction. He underwent PIPAC with no apparent complications. Rising inflammatory parameters were detected postoperatively in combination with fecal secretion from inlaid drainages. After undergoing a laparotomy and intestinal suture, his condition worsened and he died 29 days later in the intensive care unit (ICU).

#### Complications while inducing anesthesia

While the anesthesia was being induced, three patients suffered an aspiration, followed by pneumonia. During admission, one patient had presented with a mild subileus and another with a gastric outlet stenosis. After the aspiration incidents, all patients were transferred to the ICU and calculated antibiosis was initiated immediately. Nevertheless, two of these three patients died three and four days after aspiration, respectively. The other patient was treated successfully and discharged after 15 days.

#### Predicting complications

Given the relatively high incidence for non-access and intestinal injury, we evaluated ways to predict them. While we detected no significant connection between the Adhesion Score in one procedure and the risk for non-access or bowel lesion in the following PIPAC, we noticed that previous CRS with HIPEC and multiple previous abdominal surgeries served as predicting factors.

**Fig. 3:**
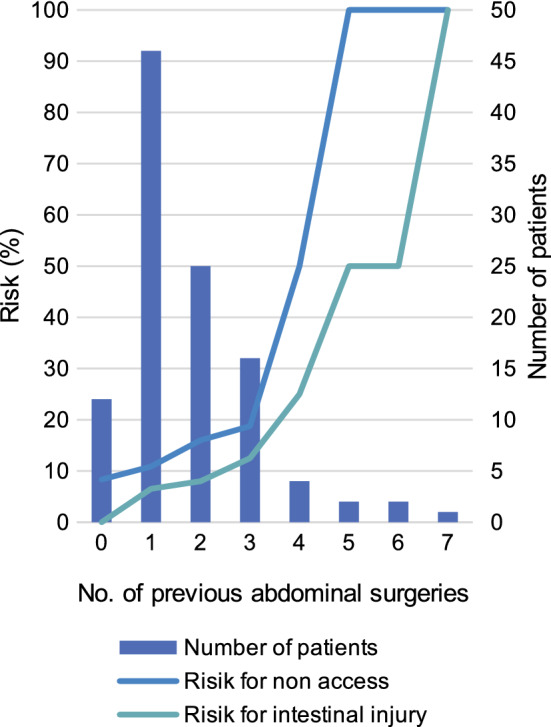
Number of patients with the respective numbers of previous abdominal surgeries (except PIPAC procedures). The risk for a non-access abdomen or intestinal injury during a PIPAC procedure rises with increasing numbers of abdominal operations. *PIPAC* pressurized intraperitoneal aerosol chemotherapy

Patients who had already undergone CRS with HIPEC carried a significantly higher risk of a non-access abdomen with an odds ratio (OR) of 5.9 (*χ*^2^, *p* < 0.01). These patients also showed a significantly higher risk of intestinal injury during surgery (OR = 6.4; *χ*^2^, *p* < 0.01). Stratifying our cohort into patients with three or more abdominal surgeries (other than PIPAC) and those with fewer yielded similar results. Patients who had three or more abdominal surgeries carried a significantly higher risk for non-access abdomen (OR = 4.9; *χ*^2^, *p* < 0.01) and bowel lesions (OR = 4.9; *χ*^2^, *p* = 0.01) (Fig. 3).

If, throughout this study, only those patients had undergone PIPAC who did not fall into the aforementioned risk groups, we would have observed only nine non-access situations (5.9%) and five intestinal injuries (3.3%). This corresponds to a potential absolute risk reduction of 3.9% (non-access) or 1.9% (intestinal injury) and a potential relative risk reduction of 66.5% (non-access) or 57.0% (intestinal injury).

### Follow-up

For the current analysis, we stopped documenting new PIPACs on the 2nd of June 2020. Follow-up was completed by the 10th of December 2020. This was necessary in 84 patients (whose disease course and—when applicable—death had not already been documented in the hospital’s patient registry). We were able to complete the follow-ups in 16 patients with the help of Leipzig’s local cancer registry. Of the 74 letters sent out to the respective physicians responsible for further treatment, 42 (57%) were answered.

By the end of this study period, only six patients were still undergoing PIPAC therapy. The most common reason for ending the treatment was patients’ death (*n* = 26, 34%). If death occurred within three months after the last scheduled PIPAC, we assumed death was the reason for ending treatment, provided no other reason had been documented. PIPAC was discontinued in twenty (26%) and six (8%) patients, because of progressing tumor disease or adhesions in the last laparoscopy, respectively. We observed regressing PSMs in 12 patients (16%), of whom seven received no further topical therapy, and five underwent CRS with HIPEC. Five patients (6%) chose not to continue PIPAC, and for eight patients (10%) no reason was documented (Supplementary Fig. 2).

Patients, whose median overall survival (OS) was the shortest, were those who had gynecological primary tumors: 136 days after their first scheduled PIPAC. The group of “others” showed the longest median OS (mesothelioma, pseudomyxoma peritonei, and CUP), namely 604 days. Kaplan–Meier analysis revealed that the median OS of all patients combined was 264 days (interquartile range 108–586) (Supplementary Table 1).

## Discussion

The efficacy and safety of PIPAC as a novel treatment for PSMs is still being investigated (Kepenekian et al. 2022). Being an “off-label” form of local therapy, PIPAC should best be carried out within a clinical-trial context. It must still be critically investigated and discussed as an “experimental” technique. In this study, symptoms, complications, and adverse events were rigorously documented. We thereby provide honest real-world evidence of this new procedure.

C and D were used as chemotherapeutic agents in the PIPAC procedure’s first description (Solaß et al. 2012, 2014), with the possibility of using oxaliplatin (OX) already mentioned. While only C and D were applied in this study, other centers (Kurtz et al. 2018; Siebert et al. 2021; Simone et al. 2020) used OX instead in patients with colorectal primary tumors. The main concern with OX is its toxicity, especially severe abdominal pain (Sgarbura et al. 2019). Recent reviews (Alyami et al. 2019a; Tempfer et al. 2018; Winkler et al. 2020) have examined this toxicity across several studies. The pooled incidences of adverse events Grade III or higher according to Common Terminology Criteria for Adverse Events (CTCAE) were 12.6% (Winkler et al. 2020), 14.8% (Alyami et al. 2019a), and 9.4% (Tempfer et al. 2018). For the latter, there was no difference between studies with or without OX. Note that these studies were very heterogeneous concerning primary tumors, sCHT, and doses used for C/D and OX, and no uniform CTCAE version was used. The recorded rate of severe complications in our study (CDC ≥ III) was only 2.7%. Keep in mind that the CDC applies to surgical complications (Dindo et al. 2004). The oncological CTCAE, on the other hand, is usually used to classify adverse events after chemotherapy. In practice, the CTCAE is used much more frequently, but inconsistently with regard to complications and adverse events (Ploug et al. 2020). Unfortunately, as the severity grades among both classifications cannot be validly compared, they should both be used in the future, each for its indication, until a new consensus to compare surgical outcomes is forthcoming (Lehmann et al. 2016).

In accordance with Hübner et al. (2017), we observed no change in mean operating time throughout this study. No learning curve was apparent in this regard. Alyami et al. (2017; 2019b), however, described a learning curve in terms of morbidity and mortality. In 2017, mortality was still 6.8% and significant complications occurred in 9.7% (CTCAE 3/4), most at the study’s beginning. Then in 2019, neither mortality nor significant complications occurred. We also noticed changes in complication rates over the course of this study, depending also on the number of surgeons. A key factor might be experience in patient selection. As described by Winkler et al. (2020), PIPAC should only be carried out in centers with a high procedure count. Patients should be strictly selected. In cases of “poor performance status, extraperitoneal disease, bowel obstruction, massive ascites, or rapidly progressive disease” (Winkler et al. 2020), PIPAC should not be recommended. Surgeons new to this procedure should first be introduced to it and intensively trained by an experienced surgeon, as practiced in our team.

Non-access is one of the main factors that limits PIPAC’s repeatability. Reviews by Grass et al. (2017), Alyami et al. (2019a), and Tempfer et al. (2018) describe a non-access rate of 0–24%, with 0% occurring only in studies with 40 or fewer procedures. Our observed non-access rate of 9.9% is in this range and even below the pooled rate of 11% (Tempfer et al. 2018). We also showed a relatively low rate of major postoperative complications, as 70% of all non-access cases had a complication lower than Grade II according to the CDC. Kurtz et al. (2018) described an association between previous CRS with HIPEC and non-access. Tempfer et al. (2015b) correlated “a high number of previous surgeries with laparoscopic non-access (*p* < 0.01)” in women with mainly ovarian cancer. To predict a non-access abdomen, we also identified two major risk factors in patient history: those, who had undergone more than two abdominal interventions, or one CRS with HIPEC in the past. In the future, we will consider these factors as contraindications for PIPAC. If a patient has already presented a non-access abdomen once in our hands, no further attempt should be made.

Anesthesia-related intra- or postoperative complications were also reported by Alyami et al. (2017) (1 of 5 deaths) and Kurtz et al. (2018) (1 aspiration without further complications). While the overall rate of Grade IV or V complications was similar in their studies and ours, the proportion of anesthesia-related events was higher in this trial. Two out of the three postoperative deaths and one of the five Grade IV events occurred after aspiration while inducing anesthesia. Despite the relatively low number of anesthesia-related complications (1.3%), particular caution while inducing anesthesia is essential, especially when the patient suffers from subileus, dysphagia, or gastric outlet stenosis.

The rise in CRP and leucocytes for a few days after PIPAC has been widely observed (Grass et al. 2017) and is usually unproblematic. No increased renal or hepatic toxicity has been reported from PIPAC (Kim et al. 2020; Larbre et al. 2018). Common sCHT effects are—as theoretical deliberations (Solaß et al. 2012) have suggested—not associated with this kind of therapy (Alyami et al. 2019a; Tempfer et al. 2018). This makes PIPAC a well-tolerated, minimally invasive alternative for patients with major side effects from sCHT.

Like many studies (Kurtz et al. 2018; Sgarbura et al. 2019; Tabchouri et al. 2021) on feasibility and effect, we have demonstrated macroscopic (PCI) and microscopic (tumor cell count) stability over multiple PIPAC procedures in conjunction with a lessening tendency. Adhesions increased (as described by Rovers et al. (2021), but no significant assertion can be made, while others (Hübner et al. 2017) reported no PIPAC impact on intraabdominal adhesions. This study confirmed PIPAC’s limiting effect on ascites volume, as described by Tempfer et al. (2015a) in patients with ovarian cancer. Other parameters indicating the quality of life and overall health status (e.g., QL2, NRS, ECOG) remained constant. These findings encourage the use of PIPAC to control PSMs, and to retain a decent quality of life.

### Limitations

As a single-center registry study, we cannot prove a causal effect of PIPAC (especially on survival). Our patient cohort was heterogeneous with regard to their primary tumors and prior treatment regimes. We measured ascites volumes intraoperatively only, not accounting for additional ascites punctures. These measurements, therefore, yield only a rough estimate of a patient’s actual ascites burden.

## Supplementary Information

Below is the link to the electronic supplementary material.

Supplementary file1 (EPS 920 KB) Frequency of symptoms recorded before each PIPAC procedure. Multiple answers are possible. PIPAC = pressurized intraperitoneal aerosol chemotherapy

Supplementary file2 (EPS 739 KB) Reasons to stop PIPAC. PIPAC = pressurized intraperitoneal aerosol chemotherapy

Supplementary file3 (DOCX 14 KB)

Supplementary file4 (DOCX 14 KB)

## Data Availability

The datasets generated during the current study are available from the corresponding author on reasonable request.
