# Supplementary material for: Pressurized intraperitoneal aerosol chemotherapy (PIPAC) in patients with peritoneal surface malignancies (PSM): a prospective single-center registry study
Source: J Cancer Res Clin Oncol. 2022 Dec 13;149(3):1331–41. doi: 10.1007/s00432-022-04517-w (PMC9984350; doi:10.1007/s00432-022-04517-w)
Supplement: Supplementary file 3 — Supplementary file3 (DOCX 14 KB) [file 432_2022_4517_MOESM3_ESM.docx]

Supplementary Table 1 Number of patients, categorized by primary tumor

| Primary | n (%) | Median OS (days) |
| --- | --- | --- |
| Gastric | 41 (38%) | 207 |
| Colorectal | 26 (24%) | 304 |
| PB^a^ | 17 (16%) | 265 |
| Gynecological^b^ | 9 (8%) | 136 |
| Others^c^ | 15 (14%) | 604 |

For each primary tumor, the median overall survival (OS) in Kaplan-Meier analysis is specified.

^a^ n = thirteen pancreatic, three bile duct, and one gall bladder cancer.

^b^ n = two breast, three ovary, and four uterine cancers.

^c^ n = seven mesotheliomas, three pseudomyxoma peritonei, and five cancers of unknown primary (CUP).

PB = pancreatico-biliary.
