# Supplementary material for: Pressurized intraperitoneal aerosol chemotherapy (PIPAC) in patients with peritoneal surface malignancies (PSM): a prospective single-center registry study
Source: J Cancer Res Clin Oncol. 2022 Dec 13;149(3):1331–41. doi: 10.1007/s00432-022-04517-w (PMC9984350; doi:10.1007/s00432-022-04517-w)
Supplement: Supplementary file 4 — Supplementary file4 (DOCX 14 KB) [file 432_2022_4517_MOESM4_ESM.docx]

Supplementary Table 2 Number of patients with or without systemic chemotherapy and with operative procedures before PIPAC

|  | n (%) |
| --- | --- |
| Systemic Chemotherapy |  |
| Started before PIPAC | 91 (84%) |
| Started together with PIPAC | 13 (12%) |
| Received between PIPAC-cycles | 54 (54%) |
| None | 4 (4%) |
| Operative procedures before PIPAC |  |
| Resection of primary | 78 (72%) |
| CRS with HIPEC | 12 (11%) |

PIPAC = pressurized intraperitoneal aerosol chemotherapy. CRS = cytoreductive surgery. HIPEC = intraperitoneal hyperthermic chemoperfusion.
